# Effective Magnetic MOFs Adsorbent for the Removal of Bisphenol A, Tetracycline, Congo Red and Methylene Blue Pollutions

**DOI:** 10.3390/nano11081917

**Published:** 2021-07-26

**Authors:** Guangpu Zhang, Rong Wo, Zhe Sun, Gazi Hao, Guigao Liu, Yanan Zhang, Hu Guo, Wei Jiang

**Affiliations:** 1 National Special Superfine Powder Engineering Research Center of China, School of Chemistry and Chemical Engineering, Nanjing University of Science and Technology, Nanjing 210094, China; gpzhang@njust.edu.cn (G.Z.); worong0614@163.com (R.W.); sunzhe97529@163.com (Z.S.); hgznjust1989@163.com (G.H.); guigao.liu@njust.edu.cn (G.L.); superfine_jw@126.com (W.J.); 2 College of Materials Science and Engineering, Nanjing Tech University, Nanjing 211816, China

**Keywords:** magnetic, metal-organic framework, adsorption, water treatment

## Abstract

A magnetic metal−organic frameworks adsorbent (Fe_3_O_4_@MIL-53(Al)) was prepared by a typical solvothermal method for the removal of bisphenol A (BPA), tetracycline (TC), congo red (CR), and methylene blue (MB). The prepared Fe_3_O_4_@MIL-53(Al) composite adsorbent was well characterized by scanning electron microscope (SEM), transmission electron microscope (TEM), X-ray diffraction (XRD), and fourier transform infrared spectrometer (FTIR). The influence of adsorbent quantity, adsorption time, pH and ionic strength on the adsorption of the mentioned pollutants were also studied by a UV/Vis spectrophotometer. The adsorption capacities were found to be 160.9 mg/g for BPA, 47.8 mg/g for TC, 234.4 mg/g for CR, 70.8 mg/g for MB, respectively, which is superior to the other reported adsorbents. The adsorption of BPA, TC, and CR were well-fitted by the Langmuir adsorption isotherm model, while MB followed the Freundlich model, while the adsorption kinetics data of all pollutants followed the pseudo-second-order kinetic models. The thermodynamic values, including the enthalpy change (Δ*H*°), the Gibbs free energy change (Δ*G*°), and entropy change (Δ*S*°), showed that the adsorption processes were spontaneous and exothermic entropy-reduction process for BPA, but spontaneous and endothermic entropy-increasing processes for the others. The Fe_3_O_4_@MIL-53(Al) was also found to be easily separated after external magnetic field, can be a potential candidate for future water treatment.

## 1. Introduction

Environmental pollution, especially water pollution caused by dyes, has drawn public and scientific attention in recent years. Exposure to hazardous organic dyes, which are widely used in the agriculture and textile industries, cause severe water shortages and ecological damages [1,2]. In addition to controlling the discharge of wastewater, it is necessary to remove as many contaminants as possible before discharge. To this end, methods have been developed for treating pollutants in water, including electrochemical [3,4,5], redox [6,7], coagulation [8,9], and photocatalytic methods [10,11]. However, these strategies require complex processes and expensive instruments and have technical limitations that require them to be combined with complementary methods to remove low concentrations of pollutants. Adsorption provides a simple, efficient and low-cost method for water treatment [10,12,13,14,15]. Traditional adsorbents, such as activated carbon, zeolites, silica microspheres and natural fibers, have been used for the adsorptive removal of pollutants; however, their applications are limited due to their insufficient adsorption capacity, relatively low thermochemical stability, and poor reproducibility [16]. Accordingly, new types of adsorbents should be developed that can remove different types of organic dye.

Metal-organic frameworks (MOFs) are crystalline materials that are formed via the self-assembly of organic ligands and metal ions. Due to their larger surface area, greater porosity, and tunable pore sizes, MOFs are popular for photocatalysis, electrocatalysis, enzyme immobilization, gas storage, and chemical sensing [17,18,19,20,21,22,23]. Recently, MOFs also have been used to treat environmental contaminants. Less-toxic MIL-53(Al){Al(OH)[O_2_C-C_6_H_4_-CO_2_]}, which has a high thermal stability, structural flexibility, and a lower-cost, has become a candidate for pollutant treatment [24,25]. One promising approach for enhancing the functionality of MOFs is the use of a magnetic composite structure [26,27]. This type of hybrid adsorbent can facilitate the recycling of MOFs after dye adsorption because they can simply be separated using an external magnetic field; therefore, preparing magnetic MOF composites has a significance for adsorption and separation of pollutants [28,29].

The solvothermal method is a typical method to prepare the metal-organic framework materials. Most MOFs are synthesized by this method. The solvothermal method is the metal ions and organic ligand dissolving in organic solvent composite material under high temperature and pressure in the reaction kettle. This method is simple to operate and can prepare the material in one step. Moreover, the crystal construction made by a solvothermal method has fewer defects. Therefore, in this work, a magnetic MOF adsorbent with a structure (Fe_3_O_4_@MIL-53(Al)) was designed and prepared by a solvothermal method and then used to remove four representative pollutants bisphenol A (BPA), tetracycline (TC), congo red (CR), and methylene blue (MB). Due to the porous structure of MIL-53(Al) and the presence of magnetic Fe_3_O_4_ particles, the Fe_3_O_4_@MIL-53(Al) composite adsorbent effectively treated wastewater and could be reused after removal by an external magnetic field. The influence of the adsorbent quantity, adsorption time, pH, ionic strength, and other factors on the adsorption was also explored. The adsorption properties of the target pollutants, including kinetics, isotherms, and thermodynamics, were also discussed.

## 2. Experimental

### 2.1. Materials

Table 1 shows a list of the empirical formula, suppliers, molecular weight and purity of the chemicals used in this work. Ferric chloride (FeCl_3_·6H_2_O), ethylene glycol, anhydrous sodium acetate (NaAc), *N*, *N*-dimethylformamide (DMF), methyl alcohol, sodium hydroxide, and hydrochloric acid were purchased from Sinopharm Chemical Reagent Co., Ltd., Beijing, China. Trisodium citrate dihydrate and aluminum nitrate nonahydrate were obtained from Xilong Scientific Co., Ltd., Guangzhou, China. 1,4-Dicarboxybenzene was purchased from Nanjing Wanqing Pharm. Co., Ltd., Nanjing, China. Bisphenol A was supplied by Shanghai Aladdin BioChem Technology Co., Ltd., Shanghai, China. Tetracycline was purchased from Nanjing Jiaozi Rattan Scientific Instrument Co., Ltd., Nanjing, China. Congo red (CR), methylene blue (MB), and sodium chloride (NaCl) were obtained from Chengdu Kelong Chemical Industry, Chengdu, China. Absolute ethyl alcohol was purchased from Nanjing Chemical Reagent Co., Ltd., Nanjing, China. All reagents and solvents were used as received without further purification.

### 2.2. Characterization

The crystal structure of the synthesized adsorbent was examined by XRD (D8 Advance, Bruker, Karlsruhe, Germany), using Cu K_α_ radiation in the range of 5–80° (2θ), voltage of 40 kV, current of 40 mA and wavelength of 1.54 Å. The specific procedure involved taking a small sample and compacting it on a glass sheet and then placing it into the instrument for analysis. Fourier-transform infrared (FT-IR) spectra were recorded in the solid-state (KBr pellet) using a Vector 22 spectrophotometer (Bruker). The spectral scanning range was 4000 cm^−1^–500 cm^−1^, the resolution was 4 cm^−1^, and the number of scans was 32. The transmission electron microscope (TEM) was carried out to observe the particle size and morphology. The specific procedure involved taking a small amount of prepared samples and dispersing them in anhydrous ethanol with a toothpick. Then, samples were ultrasonicated to fully disperse the particles, and then a few drops of suspension were extracted with a dropper and dropped them into a copper grid. TEM images were obtained after the liquid evaporated. The size and morphology of Fe_3_O_4_@MIL-53(Al) were also observed with a Scanning electron microscope (SEM) EM (Model-S480 II FESEM). The specific procedure involved extracting a few dried samples with a toothpick and sticking them on a conductive adhesive. After spraying in gold, they were placed into the SEM for analysis. The magnetic saturation strength of the samples was tested by vibrating sample magnetometry (VSM) (LakeShore 735) with the test magnetic moment range of −20–20 kOe. The specific procedure is as follows: take about 20 mg sample and put it into a plastic tube, plug the two ends with cotton, and then put it into the instrument for testing. A UV/Vis spectrophotometer (Agilent Cary 100 UV-2600, Santa Clara, CA, USA) was used to measure the dye concentrations in the solution. The spectral range was 200–800 nm.

### 2.3. Preparation of Fe_3_O_4_@MIL-53(Al)

**Scheme 1:**
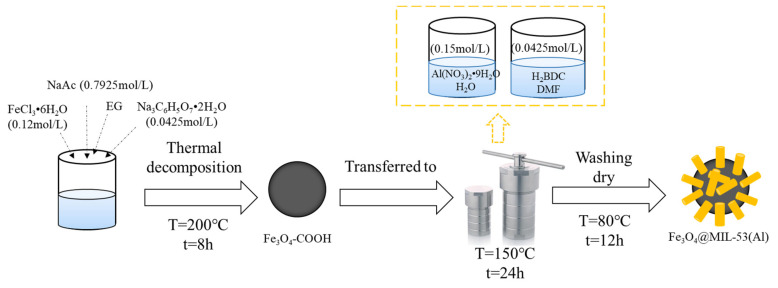
Illustration of the Preparation Process of Fe_3_O_4_@MIL-53(Al) (T = Temperature; t = time).

As shown in Scheme 1, preparation of carboxylated Fe_3_O_4_-COOH particles was carried out as follows. FeCl_3_·6H_2_O (1.3 g, 4.8 mmol) and Trisodium citrate dihydrate (0.5 g, 1.7 mmol) were dissolved in 40 mL ethylene glycol with ultrasonic stirring for 10 min. Then, sodium acetate (2.6 g, 31.7 mmol) was added and ultrasonication was performed for another 30 min. The solution was then transferred to a polytetrafluoroethylene reactor and was reacted at 200 °C for 8 h. After that, the solution was cooled to an ambient temperature, and the prepared materials were magnetically separated by an external magnetic field, washed alternately with deionized water and ethanol, and dried at 60 °C. The product, carboxylated Fe_3_O_4_-COOH particles, was obtained.

Magnetic MOFs (Fe_3_O_4_@MIL-53(Al)) were obtained by the following procedure: Aluminum nitrate nonahydrate (0.5625 g, 1.5 mmol) and 1,4-dicarboxybenzene (0.28 g, 1.7 mmol) were dissolved in 10 mL deionized water and 40 mL N, *N*-dimethylformamide (DMF), respectively. After complete dissolution, these two solutions were ultrasonically mixed for 30 min. After that, Fe_3_O_4_-COOH (0.1 g, 0.4 mmol) was added to the solution, and the particles were evenly dispersed by ultrasonication for another 10 min. The solution was then transferred to the Polytetrafluoroethylene (PTFE) reactor and reacted at 150 °C for 24 h. At the end of the reaction, the solution was activated with *N*, *N*-dimethylformamide for 8 h at 130 °C After activation, the product was separated by an external magnetic field and washed three times with methanol. Then, the solid was dried at 80 °C for 12 h, and the magnetic MOF with core/shell particles (Fe_3_O_4_@MIL-53(Al)) was obtained.

### 2.4. Adsorption Experiments

In the adsorption experiments, BPA, TC, CR, and MB were dissolved in deionized water to make solutions with different concentrations (20–150 mg L^−1^). The adsorbents were added to 20 mL of a 100 mg L^−1^ dye solution, and then shaken at 500 rpm in a constant-temperature oscillator. The ionic strength was modulated by adding different masses of sodium chloride. The pH values (5.0–11.0) were adjusted by 0.1 M HCl and 0.1 M NaOH solutions. The supernatant was measured by a UV-Vis spectrophotometer. All parallel experiments were performed in triplicate to ensure accuracy, and the average results were employed for further data analysis. The removal efficiency of dyes and the adsorption amount were calculated by the following formula:

$$Dye\;removal\;efficiency\;\left(\%\right)=\frac{M_{0}-M}{M_{0}}$$

$$Q_{e}=\frac{\left(C_{0}-C_{e}\right)V}{m}$$

where *M*_0_ (g) is the initial mass of the dye, and *M* (g) is the mass of the dye after adsorption; *Q*_e_ (mg g^−1^) is the equilibrium dye adsorption amount; *V* (mL) is the volume of the dye solution; *m* (mg) is the adsorbent mass.

Based on adsorption experiments, we discuss the adsorption kinetics models, adsorption isotherm and adsorption thermodynamics to explore the possible adsorption mechanism between adsorbent and dyes. A detailed calculation process was listed in the Supplementary Materials.

### 2.5. Regeneration Experiments

After each adsorption, Fe_3_O_4_@MIL-53(Al) was alternately eluted by 0.1 M NaOH and ethanol solution until no dye could be detected in the supernatant. The regenerated Fe_3_O_4_@MIL-53(Al) was dried in a vacuum oven at 60 °C overnight.

## 3. Results and Discussion

### 3.1. Characterization of Fe_3_O_4_@MIL-53(Al)

**Figure 1:**
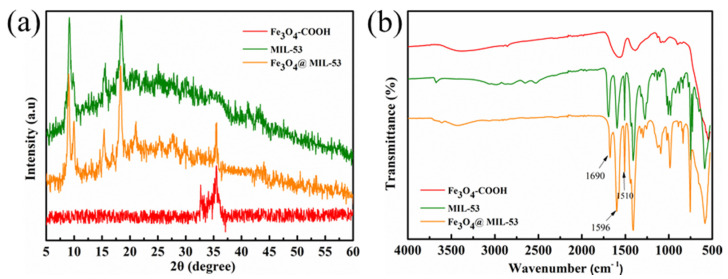
(**a**) XRD pattern of Fe_3_O_4_-COOH, MIL-53(Al) and Fe_3_O_4_@MIL-53(Al); (**b**) FT-IR spectra of Fe_3_O_4_-COOH, MIL-53(Al) and Fe_3_O_4_@MIL-53(Al).

Figure 1a shows the XRD patterns of Fe_3_O_4_-COOH, MIL-53(Al), and Fe_3_O_4_@MIL-53(Al). Both Fe_3_O_4_-COOH and Fe_3_O_4_@MIL-53(Al) showed characteristic diffraction peaks of Fe at 35.5°, indicating the existence of Fe in Fe_3_O_4_@MIL-53(Al). Meanwhile, MIL-53 (Al) peaks at 8.9°, 10.15°, 15.3°, 18.38° and 25.5°, were consistent with those in the literature [30], and confirming that the product was MIL-53 (Al). The XRD pattern of Fe_3_O_4_@MIL-53(Al) has a diffraction peak at 8.9°, 10.15°, 15.3°, 18.38°, 25.5°, and 35.5°, which includes the characteristic peak of Fe, and also the characteristic peaks of MIL-53(Al), indicating that Fe_3_O_4_@MIL-53(Al) was composed of Fe_3_O_4_ and MIL-53(Al). 

The infrared spectra of Fe_3_O_4_-COOH, MIL-53(Al), and Fe_3_O_4_@MIL-53(Al) are shown in Figure 1b. In the spectra of Fe_3_O_4_-COOH and Fe_3_O_4_@MIL-53(Al), the peak at 553 cm^−1^ was related to the Fe-O vibrational bond. The peak of MIL-53(Al) and Fe_3_O_4_@MIL-53(Al) at 1000–1100 cm^−1^ was due to the presence of Al-O bonds, and the absorption peaks at 1596 and 1510 cm^−1^ were due to the asymmetric stretching vibration of C-O. The absorption peak at 1690 cm^−1^ was related to the stretching vibration of –C=O [31]. The Fe_3_O_4_@MIL-53(Al) particles synthesized from MIL-53(Al) and Fe_3_O_4_-COOH contained absorption peaks at 553 cm^−1^, and also retained characteristic peaks at 1690, 1596, and 1510 cm^−1^, indicating that the addition of MIL-53(Al) enriched the functional groups of the composite particles Fe_3_O_4_@MIL-53(Al), thus proving the successful preparation of the Fe_3_O_4_@MIL-53(Al) composite particles.

**Figure 2:**
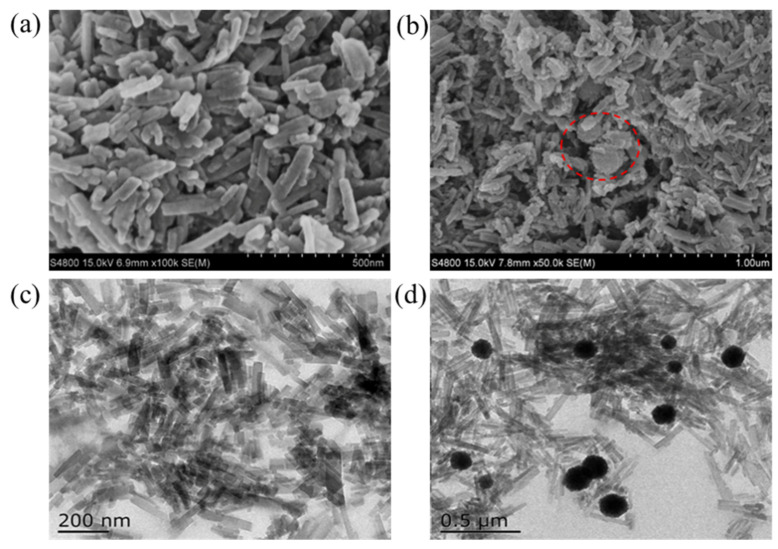
SEM images of (**a**) MIL-53(Al), (**b**) Fe_3_O_4_@MIL-53(Al); TEM images of (**c**) MIL-53(Al) and (**d**) Fe_3_O_4_@MIL-53(Al).

The morphology, structure, and size of Fe_3_O_4_@MIL-53(Al) are shown in Figure 2. In the SEM image, MIL-53(Al) appears as thick and short rods stacked (200 nm in length and 20 nm in diameter) in Figure 2a. After the addition of Fe_3_O_4_ particles, we still observed much the short rod-like morphology and few spheres (*ca* 300 nm in diameter, the red dotted line part) in Figure 2b. These exposed spherical spheres were Fe_3_O_4_ that were not completely covered by MIL-53(Al). On the one hand, this proves the existence of Fe_3_O_4_ in the Fe_3_O_4_@MIL-53 (Al) composite particles; on the other hand, this also indicates that most Fe_3_O_4_ was completely covered by MIL-53(Al) and lies inside the Fe_3_O_4_@MIL-53(Al) composite, which is not easily observed. The same conclusion was obtained by TEM in Figure 2c,d. From the TEM image, the stacked MIL-53 (Al) are baculiform and pure particles before adding Fe_3_O_4_. After complexing with Fe_3_O_4_, the Fe_3_O_4_ particles are black and spherical, distributing inside the MIL-53 (Al) to form composite particles. The black particles range from 100 to 300 nm in diameter and most of them are completely surrounded by MIL-53 (Al).

**Figure 3:**
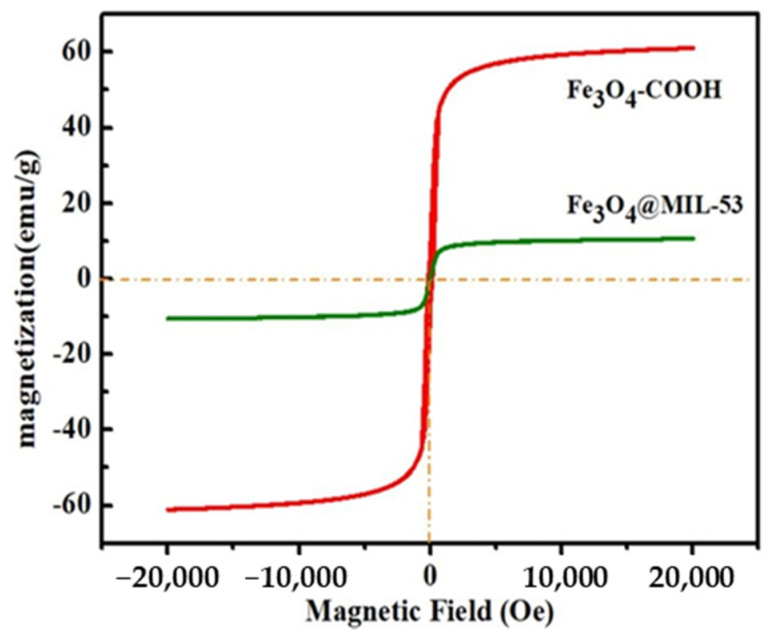
Vibrating sample magnetometry image of Fe_3_O_4_-COOH and Fe_3_O_4_@MIL-53(Al).

The magnetic performance was measured by magnetic hysteresis loops with varying magnetic fields (Figure 3). Fe_3_O_4_-COOH and Fe_3_O_4_@MIL-53(Al) are both magnetic with magnetic saturation strengths of 61.12 emu/g and 10.61 emu/g, respectively. Compared with the pure Fe_3_O_4_-COOH particles, the magnetism of composite particle Fe_3_O_4_@MIL-53(Al) becomes weaker. Because MIL-53(Al) is a non-magnetic material, the introduction of non-magnetic materials weakened the magnetic properties of the composite. Although the magnetism of Fe_3_O_4_@MIL-53(Al) is inferior to pure Fe_3_O_4_-COOH, the Fe_3_O_4_@MIL-53(Al) still is sedimentation and separate rapidly with an external magnetic field, which is convenient for recycling.

### 3.2. Adsorption of BPA, TC, CR, and MB

#### 3.2.1. Influence of Adsorbent Amounts

**Figure 4:**
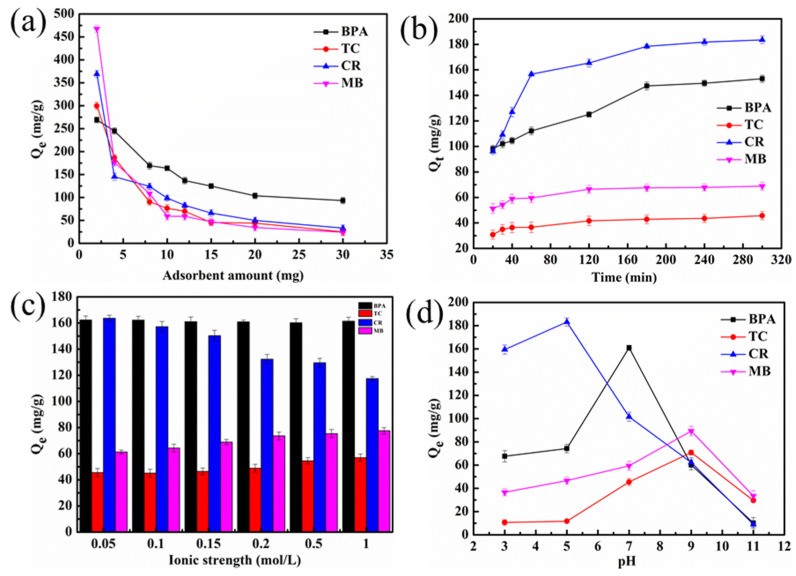
(**a**) influence of adsorbent amounts on BPA, TC, CR, and MB; (**b**) influence of time on BPA, TC, CR, and MB; and (**c**) influence of ionic strength on BPA, TC, CR, and MB; (**d**) Influence of pH on BPA, TC, CR, and MB. Those error bars are standard deviations. The materials were independently prepared to repeat the same experiment at least three times to ensure the accuracy of the data.

The adsorption capacity of bisphenol A, tetracycline, congo red, and methylene blue all decreased upon increasing the Fe_3_O_4_@MIL-53(Al) absorbent amount, which is illustrated in Figure 4a. In the beginning, the adsorbents can provide plentiful and accessible unsaturated active sites at the low amount adsorbents. However, with the increase of the dosage of adsorbent, there were more adsorption sites that are not occupied for the adsorption process, leading to a reduction in the adsorption capacity. Therefore, in subsequent experiments, the adsorbent mass used for bisphenol A and tetracycline was 0.6 g/L and 0.75 g/L, respectively, and the addition amounts of Congo red and methylene blue were 0.2 g/L and 0.5 g/L, respectively.

#### 3.2.2. Influence of Adsorption Time

In this section, the effect of adsorption time was investigated from 20 to 300 min. According to Figure 4b, upon extending the adsorption time, the adsorption capacity of bisphenol A and Congo red both increased. At 0–180 min, the adsorption capacity of these two pollutants increased rapidly. From 180 to 300 min, the adsorption capacity of bisphenol A and Congo red did not increase significantly upon the increasing time, so 180 min was the adsorption equilibrium time of bisphenol A and Congo red; however, the adsorption capacity of tetracycline and methylene blue did not change significantly with time after 120 min, so 120 min was the optimal adsorption equilibrium time for tetracycline and methylene blue.

Although the adsorption equilibrium time of different pollutants was different, the adsorption process was generally divided into two stages. The first stage is the rapid adsorption stage, in which the adsorption amount of pollutants increased significantly with time. The second stage is the slow adsorption stage. Even if the adsorption time increases, the adsorption amount of pollutants increased slowly, and the final equilibrium adsorption amount did not change. This is because, at the initial stage of adsorption, a large number of holes and adsorption sites on the surface and inside of the adsorbent can be occupied by contaminant molecules; however, upon extending the time, pollutant molecules occupied the pores and adsorption sites, and the available vacancy sites became less and less, leading to a slower adsorption rate before finally reaching an adsorption equilibrium [32].

#### 3.2.3. Influence of Ionic Strength

The influence of ionic strength on the adsorption efficiency was investigated by varying the NaCl concentration from 0.01 to 1.0 M. Figure 4c shows that different pollutants are affected by the ion intensity to different degrees. Among them, bisphenol A was less affected by ions. Upon changing NaCl concentration, the adsorption amount of bisphenol A changed only slightly. For Congo red, the effect of ionic strength was negative and the adsorption capacity decreased upon increasing the ionic strength. When the NaCl concentration increased from 0.05 to 1 M, the adsorption capacity of Congo red decreased from 163 mg/g to 117 mg/g. Obviously, the higher the ionic strength, the more unfavorable the adsorption of Congo red by Fe_3_O_4_@MIL-53(Al). For Fe_3_O_4_@MIL-53(Al) the adsorption of tetracycline and methylene blue, the effect of ionic strength was positive. In other words, upon increasing the ionic strength, the adsorption capacity of tetracycline and methylene blue increased, indicating that ionic strength was conducive to the adsorption of tetracycline and methylene blue by Fe_3_O_4_@MIL-53(Al). When more ions were present in the solution, the adsorption capacity of tetracycline and methylene blue was more favorable.

The different effects of ion strength on adsorption are due to the different effects of ions in different adsorption systems. Generally speaking, the effect of ionic strength on adsorption is mainly reflected in two aspects; the salting-out effect and the competition effect [33,34,35,36]. Salting out was dominant in the Fe_3_O_4_@MIL-53(Al) adsorption system for tetracycline and methylene blue, because the adsorption capacities increased on increasing the ionic strength. The adsorption process of Fe_3_O_4_@MIL-53(Al) was dominated by competition because the adsorption amount of Congo red decreased after increasing the ionic strength; however, during the adsorption of bisphenol A, the salting-out effort and competition canceled each other out, or their two influences were relatively weak, and ions in the solution simply did not affect adsorption. In this case, the adsorption capacity of Fe_3_O_4_@MIL-53(Al) for bisphenol A did not change significantly upon changing the ion strength.

#### 3.2.4. Influence of pH

The pH of the solution can greatly influence the adsorption of dyes. In this work, the influence of sample pH on the removal efficiency was investigated in the pH range of 5–11, and the results are illustrated in Figure 4d. The adsorption capacity of bisphenol A reached its maximum at pH = 7. Upon increasing the alkalinity of the solution, the adsorption capacity of bisphenol A decreased sharply. This is because at pH > 9, bisphenol A was ionized into the monovalent anion HBPA^-^ and bivalent anion BPA^2−^, which electrostatically rejected the adsorbent due to the negative surface charge, making the adsorption amount lower. When the solution was acidic, a large number of H^+^ and positively-charged adsorbents on the surface electrostatically repelled bisphenol A, so the adsorption capacity of bisphenol A reached the maximum when the pH was 7. However, Congo red is favorable for adsorption under acidic conditions. When the pH was 3–5, the adsorption capacity of Fe_3_O_4_@MIL-53(Al) for Congo red increased upon increasing the pH. When the pH was between 5 and 11, the adsorption capacity of Congo red decreased upon increasing the pH, so the adsorption effect of Congo red was the best at pH = 5. This is because when pH < 6, there is an electrostatic attraction between the anionic dye Congo red and the positively charged adsorbent, which increases the adsorption capacity. However, when the pH > 6, the structure of Congo red changes and the surface becomes negatively charged. Upon increasing the pH, the charge on the surface of the adsorbent becomes negative, and the electrostatic repulsion reduces the amount of adsorption.

However, the adsorption capacity of Fe_3_O_4_@MIL-53(Al) for tetracycline and methylene blue increased when the pH was increased and reached the maximum at pH = 9. When the pH of the solution was further increased to pH = 11, the adsorption capacity of Fe_3_O_4_@MIL-53(Al) for tetracycline and methylene blue was extremely low. Under acidic conditions, both tetracycline (TC^+^) and methylene blue exist as cations, which are electrostatically repelled by the positively charged adsorbent surface, resulting in a low adsorption capacity. When pH = 11, tetracycline and methylene blue are ionized as anion TC^2−^, and are electrostatically repelled by the negatively charged surface, resulting in a low adsorption capacity. When pH = 9, a similar situation would be expected, but that was not observed in the experimental results. This indicates that the primary interaction between the adsorbent and the adsorbent are not charges, but rather H bonding and π-π conjugation; therefore, the adsorption effect of Bisphenol A was best when pH = 7. The adsorption effect of Congo red was the best when pH = 5. The adsorption of both tetracycline and methylene blue was most favorable at pH = 9.

### 3.3. Adsorption Kinetics

Adsorption kinetics are used to elucidate the adsorption behaviors including mass transfer, chemical reaction, and the rate-determining step of adsorption. In this section, the pseudo-first-order, pseudo-second-order, and intraparticle diffusion models were applied to the experimental data.

**Figure 5:**
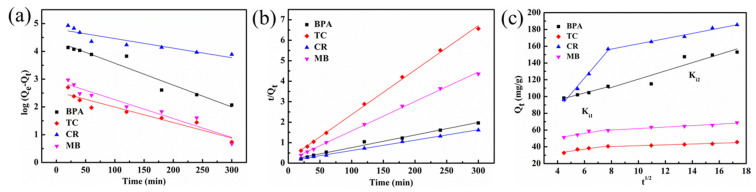
Fit of kinetic data to (**a**) the pseudo-first-order model, (**b**) the pseudo-second-order model, and (**c**) the intraparticle diffusion model on BPA, TC, CR, and MB.

The linear regression curves of the three models for adsorbing BPA, TC, CR, and MB are presented in Figure 5a–c, and all model parameters are shown in Table 2. The adsorption data of the prepared adsorbents on bisphenol A, tetracycline, Congo red, and methylene blue were better fitted by the quasi-second-order model, because the fitting coefficients were all above 0.98, and a good linear fit was obtained. The secondary dynamic adsorption equilibrium amounts of bisphenol A (BPA), tetracycline, Congo red, and methylene blue were calculated to be 162.6, 46.1, 200.0, and 69.9 mg/g. These were not far from the experimental value, indicating that the adsorption kinetics of these four pollutions follow the second-order kinetics model and process via chemical adsorption. The adsorption capacity of Fe_3_O_4_@MIL-53(Al) is associated with its number of available active sites [37,38]. In addition to the poor quasi-first-order kinetic fitting coefficient of Congo red, the *R*^2^ values of bisphenol A, tetracycline and methylene blue were all above 0.9, and the fitting coefficient was also good, indicating that bisphenol A, tetracycline, and methylene blue underwent physical adsorption on Fe_3_O_4_@MIL-53(Al).

As can be seen from *Q*t in Figure 5c, the intramolecular diffusion models of bisphenol A, tetracycline, Congo red and methylene blue do not pass through the origin, indicating that during adsorption of these four pollutants, internal diffusion is not the only step that controls adsorption, and there are two or more diffusion mechanisms that affect adsorption. The line is divided into a first stage (*K*_i1_) and second stage (*K*_i2_). The first stage involves the migration of pollutant molecules from the solution to the outer surface of the adsorbent. During the second stage, the pollutant molecules diffuse to the adsorbent pores. All four pollutants have high *R*^2^ in the second stage, indicating that the diffusion of pollutant molecules into the adsorbent pores is the rate-controlling step of these four pollutants [39].

### 3.4. Adsorption Isotherms

**Figure 6:**
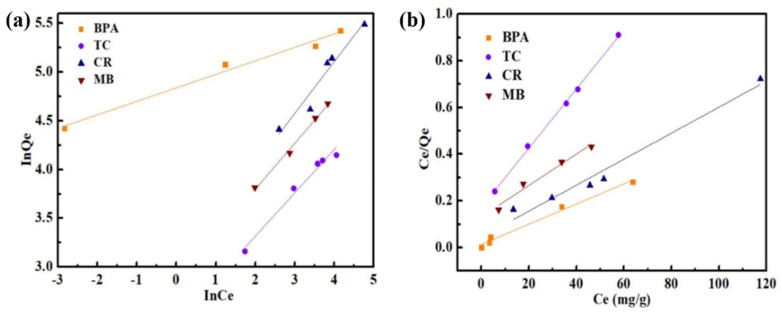
Adsorption isotherm curves of BPA, TC, CR, and MB on Fe_3_O_4_@MIL-53(Al). (**a**) Freundlich model; (**b**) Langmuir model.

The adsorption isotherm is important for investigating the adsorption type and strength between the adsorbents and the target compounds in the adsorption batch system. As can be seen from Figure 6 and Table 3, the Langmuir linear fitting coefficients of bisphenol A, tetracycline, and CR were higher than that of the Freundlich model, indicating that the Langmuir model better described the adsorption process and that Fe_3_O_4_@MIL-53(Al) adsorbed bisphenol A, tetracycline, and CR in a homogeneous and single-molecular layer process. The experimental data of the adsorption of methylene blue by Fe_3_O_4_@MIL-53(Al) was better fitted by the Freundlich isotherm model (*R*^2^ > 0.99), indicating that Fe_3_O_4_@MIL-53(Al) adsorbed methylene blue via heterogeneous, multi-molecular layer adsorption. According to the Freundlich isotherm model, the 1/*n* values of the four pollutants were all less than 1, indicating that Fe_3_O_4_@MIL-53(Al) was favorable and easily adsorbed bisphenol A, tetracycline, CR, and MB [40]. A Langmuir model was used to calculate the maximum adsorption capacity of Fe_3_O_4_@MIL-53(Al) to bisphenol A, tetracycline, Congo red, and methylene blue, which were respectively 205.00, 78.93, 179.53, and 148.81 mg/g, showing an excellent adsorption effect.

The comparison of the adsorbent developed in this work with previously reported adsorbents for the removal of BPA, TC, CR, and MB is summarized in Table 4. Although Fe_3_O_4_@MIL-53(Al) has a lower adsorption capacity than pure MIL-53(Al) or MIL-53(Fe), the functionalization with magnetic particles endows materials the ability which can be easily separated by a magnetic field. The adsorption capacities for the targeted pollutants can be compared to some reported adsorbents, verifying it to act as a potential purifying agent for removing cationic dyes from wastewater.

### 3.5. Adsorption Thermodynamics

In order to further explore the type of adsorption of bisphenol A (BPA), tetracycline, Congo red, and methylene blue adsorption by Fe_3_O_4_@MIL-53(Al) and explore the possible adsorption mechanism, the adsorption enthalpy change (Δ*H*°), the Gibbs free energy change (Δ*G*°), and entropy (Δ*S*°) were calculated (calculation process in the Supplementary Materials), and the results are listed in Table 5.

In this system, Δ*H*° > 0 indicates that the removal efficiencies of the dyes increase with an increase in temperature, and the adsorption process is an endothermic process. According to a previous report, adsorption is physical adsorption when Δ*G*° ranges from −20 to 0 kJ mol^−1^. When the Δ*G*° value ranges from −400 to −80 kJ mol^−1^, adsorption proceeds via chemical adsorption [47].

Table 5 shows that Δ*H*° for the adsorption of tetracycline, methylene blue, and Congo red by Fe_3_O_4_@MIL-53(Al) is greater than 0, and Δ*G*° is less than 0, indicating a spontaneous endothermic process. However, for the absorption process of bisphenol A, Δ*H*° and Δ*G*° were less than 0, which indicates a spontaneous exothermic process. In addition, for bisphenol A, tetracycline, methylene blue, and Congo red, |Δ*H*°| is within the range of 0–84 kJ/mol, and Δ*G*° is in the range −20 to 0 kJ/mol, which shows that the adsorption of the four pollutants was mainly physical adsorption. The Δ*G*° of bisphenol A, tetracycline, methylene blue, and Congo red increased with the temperature, showing that at a higher temperature is beneficial for the adsorption of these pollutants by Fe_3_O_4_@MIL-53(Al).

The entropy change (Δ*S*°) is also an important thermodynamic parameter. Δ*S*° > 0 indicates an increase in entropy, while Δ*S*° < 0 indicates a decrease in entropy during the adsorption system [48]. Δ*S*° is determined by solute adsorption (degree of freedom, entropy decreasing) and solvent desorption (degree of freedom increase, increase in entropy).

Table 5 shows that the Δ*S*° for the bisphenol A is different from the other three pollutants. During bisphenol A adsorption, Δ*S*° < 0, which indicates a decrease in entropy reduction and system chaos reduction. In this adsorption process, more solute molecules were adsorbed and the degree of freedom decreased, indicating an entropy-reduction process. The adsorption of the other three pollutants involved an increase in entropy, because more water molecules were desorbed from the surface of Fe_3_O_4_@MIL-53(Al) than the adsorbed amount of these three pollutants. More water molecules were desorbed from the adsorbent surface than adsorbent molecules. During the solvent desorption, an increase in entropy was dominant, which increased the entropy of the system. 

Based on Δ*H*°, Δ*G*°, and Δ*S*°, we concluded that Fe_3_O_4_@MIL-53(Al) adsorbed bisphenol A via a spontaneous, exothermic, and entropy-reduction process, which is physical adsorption; the adsorption of tetracycline, Congo red, and methylene blue was a spontaneous, endothermic and entropy-increasing process, which mainly proceeded via physical adsorption.

### 3.6. Adsorption Mechanism

After researching kinetics, isotherm and thermodynamics, we found that the adsorption of the BPA, TC, CR, and MB was all physical adsorption predominantly, which was related to the pore construction between adsorbents and pollutants. When the channels of MIL-53(Al) are occupied by guest molecules or organic ligands, the MIL-53(Al) will change its structure to possess a unique reversible property according to the adjustment of temperature or guest molecules. This also features the MIL-53(Al), or the “breathing” effect of the framework. Therefore, the Fe_3_O_4_@MIL-53(Al) have superior adsorption performances for different target pollutants. The Fe_3_O_4_@MIL-53(Al) can adjust their construction to adapt to diverse pollutant molecular sizes. Particularly noteworthy is the chemical adsorption which also exists between the BPA, TC, CR, and MB and Fe_3_O_4_@MIL-53(Al). This is due to the π-π interaction between the benzene ring that is present in both the target pollutant and the adsorbent. Moreover, the hydrogen bonding makes contributions to better adsorption performances.

## 4. Conclusions

In this work, a functionalized MOF with magnetic particles (Fe_3_O_4_@MIL-53(Al)) was designed and prepared for the removal of four pollutants. The structure and performance of Fe_3_O_4_@MIL-53(Al) were characterized by SEM, TEM, FT-IR and XRD. The maximum adsorption capacities for BPA, TC, CR, and MB were determined to be 160.9 mg/g, 47.8 mg/g, 234.4 mg/g, and 70.8 mg/g, respectively, which are much higher than that of other reported adsorbents. The adsorption of BPA, TC, and CR were well-fitted by the Langmuir adsorption isotherm model, while MB followed the Freundlich model, while the adsorption kinetics data of all pollutants followed the pseudo-second-order kinetics models. The thermodynamic values, including the enthalpy change (Δ*H*°), the Gibbs free energy change (Δ*G*°), and entropy change (Δ*S*°), showed that the adsorption processes were spontaneous and exothermic entropy-reduction for BPA, but spontaneous and endothermic entropy-increasing processes for the others. Moreover, Fe_3_O_4_@MIL-53(Al) can be easily separated by an external magnetic field due to the presence of the Fe_3_O_4_ magnetic particle. All of the results demonstrate that Fe_3_O_4_@MIL-53(Al) is a promising adsorbent for removing dyes from wastewater.

## Figures and Tables

**Table 1:** Empirical Formula, CAS Registry Number, Suppliers, and Purity of the Chemicals.

Component	Chemicals	Molecular Weight(g/mol)	Suppliers	Purity
Ferric chloride	FeCl_3_·6H_2_O	270.3	Sinopharm Chemical Reagent Co., Ltd.	A.R
ethylene glycol	C_2_H_6_O_2_	62.1	Sinopharm Chemical Reagent Co., Ltd.	A.R
anhydrous sodium acetate	CH_3_COONa	82.1	Sinopharm Chemical Reagent Co., Ltd.	A.R
Trisodium citrate dihydrate	Na_3_C_6_H_5_O_7_·2H_2_O	294.1	Xilong Scientific Co., Ltd.	A.R
N, N-dimethylformamide	C_3_H_7_NO	73.1	Sinopharm Chemical Reagent Co., Ltd.	A.R
aluminum nitrate nonahydrate	H_18_AlN_3_O_18_	375.1	Xilong Scientific Co., Ltd.	A.R
1,4-Dicarboxybenzene	C_8_H_6_O_4_	166.1	Nanjing Wanqing Pharm. Co., Ltd.	A.R
methyl alcohol	CH_3_OH	32.1	Sinopharm Chemical Reagent Co., Ltd.	A.R
Bisphenol A	C_15_H_16_O_2_	228.3	Aladdin BioChem Technology Co., Ltd.	G.C
Tetracycline	C_22_H_24_N_2_O_8_	444.4	Nanjing Jia Nanjing Jiaozi Rattan Scientific ozi Rattan Scientific Instrument Co., Ltd.	C.P
Congo red	C_32_H_22_N_6_Na_2_O_6_S_2_	696.7	Chengdu Kelong Chemical Industry	A.R
methylene blue	C₁₆H₁₈ClN₃S	319.8	Chengdu Kelong Chemical Industry	A.R
sodium chloride	NaCl	58.4	Chengdu Kelong Chemical Industry	A.R
sodium hydroxide	NaOH	40.0	Sinopharm Chemical Reagent Co., Ltd.	A.R
hydrochloric acid	HCl	36.5	Sinopharm Chemical Reagent Co., Ltd.	A.R
deionized water	H_2_O	18.0	Laboratory provision	A.R
Absolute ethyl alcohol	C_2_H_6_O	46.1	Nanjing Chemical Reagent Co., Ltd.	A.R

Note: A.R is Analytical Reagent; G.C is Gas Chromatographically pure; C.P is Chemically Pure.

**Table 2:** Parameters of Pseudo-First-Order, Pseudo-Second-Order, and Intraparticle Diffusion Models.

Kinetics	Parameters	Dyes			
		BPA	TC	CR	MB
	*Q*_cal_^a^ (mg/g)	162.6	46.1	200.0	69.9
Pseudo-Second-Order Model	*Q*_exp_^b^ (mg/g)	160.9	47.8	234.4	70.8
	*k*_2_ (g/mg/min)	2.62 × 10^−4^	2.35 × 10^−3^	2.26 × 10^−4^	1.54 × 10^−3^
	*R* ^2^	0.9897	0.9984	0.9988	0.9986
	*Q*_cal_^a^ (mg/g)	78.1	12.8	122.0	18.9
Pseudo-First-Order Model	*k*_1_ (min^−1^)	0.0079	0.0055	0.0034	0.0068
	*R* ^2^	0.9332	0.9047	0.8343	0.9236
	*k*_i1_ (mg/g/min^1/2^)	2.65	2.28	18.79	1.75
	*C*_1_ (mg/g)	87.9	23.5	18.8	45.2
Intraparticle Diffusion Models	*R* ^2^	0.8657	0.8971	0.9801	0.8249
	*k*_i2_ (mg/g/min^1/2^)	1.43	0.50	3.11	0.82
	*C*_2_ (mg/g)	127.9	36.4	131.7	53.9
	*R* ^2^	0.9329	0.9129	0.9845	0.8599

^a^*Q*_e,exp_ is the equilibrium adsorption capacities according to the experimental results. ^b^ *Q*_e,cal_ is determined by the linear fitting from the kinetic models.

**Table 3:** Isotherm Parameters of Fe_3_O_4_@MIL-53(Al) for BPA, TC, CR, and MB Adsorption.

Dyes	Langmuir Model			Freundlich Model		
	*Q*_max_ (mg/g)	*K*_L_ (L/mg)	*R* ^2^	1/*n*	*K*_F_	*R* ^2^
BPA	205.0	0.3077	0.9835	0.1380	126.2	0.9758
TC	78.9	0.0735	0.9978	0.4441	11.4	0.9726
CR	179.5	0.1253	0.9670	0.5303	19.8	0.9262
MB	148.8	0.0511	0.9611	0.4711	17.4	0.9924

**Table 4:** Comparison of the Fe_3_O_4_@MIL-53(Al) uptake with some previous works.

Adsorbent	*Q*_max_ (mg/g)	Target Pollutant	Reference
Fe_3_O_4_@MIL-53(Al)	160.9 mg/g	BPA	This Work
	47.8 mg/g	TC	
	234.4 mg/g	CR	
	70.8 mg/g	MB	
MIL-53(Al)	329.2 ± 16.5 mg/g	BPA	[31]
MIL-53(Fe)	247.7 mg/g	TC	[41]
	1 482 mg/g	CR	[42]
MIL-101-NH_2_	Less than 100 mg/g	BPA	[43]
CWs Ben-Rm-Ps ceramsite	2.56 mg/g	TC	[44]
CDGO nanosheets	23.1 mg/g	BPA	[45]
HP-β-CD	186.9 ± 7.9 mg/g	MB	[46]

**Table 5:** Thermodynamic Parameters for BPA, TC, CR, and MB Adsorption on Fe_3_O_4_@MIL-53(Al).

Dyes	*T* (K)	Δ*G°* (kJ/mol)	Δ*H°* (kJ/mol)	Δ*S°* (J/mol/K)
BAP	298	−8.23	-	-
	308	−9.04	−34.1	−81.5
	318	−9.86	-	-
TC	298	−2.62	-	-
	308	−3.37	19.7	74.9
	318	−4.12	-	-
CR	298	−3.67	-	-
	308	−6.07	67.8	240.0
	318	−8.47	-	-
MB	298	−2.48	-	-
	308	−2.96	11.8	47.9
	319	−3.44	-	-

## Supplementary Materials

The following are available online at https://www.mdpi.com/article/10.3390/nano11081917/s1. Supplementary Materials: 1. Adsorption model.
