# Supplementary material for: Effective Magnetic MOFs Adsorbent for the Removal of Bisphenol A, Tetracycline, Congo Red and Methylene Blue Pollutions
Source: Nanomaterials (Basel). 2021 Jul 26;11(8):1917. doi: 10.3390/nano11081917 (PMC8398004; doi:10.3390/nano11081917)
Supplement: Supplementary file 1 [file nanomaterials-11-01917-s001.zip › nanomaterials-1249109-supplementary.pdf]

Supplementary Materials

Effective Magnetic MOFs Adsorbent for the Removal of Bisphenol A, Tetracycline, Congo Red and Methylene Blue Pollutions

Guangpu Zhang [1], Rong Wo [1], Zhe Sun [1], Gazi Hao [1], Guigao Liu [1], Yanan Zhang [2,*], Hu Guo [1,*] and Wei Jiang [1]

1 National Special Superfine Powder Engineering Research Center of China, School of Chemistry and Chemical Engineering, Nanjing University of Science and Technology, Nanjing 210094, China; gpzhang@njust.edu.cn (G.Z.); worong0614@163.com (R.W.); sunzhe97529@163.com (Z.S.); hgznjust1989@163.com (G.H.); guigao.liu@njust.edu.cn (G.L.); superfine_jw@126.com (W.J.)

2 College of Materials Science and Engineering, Nanjing Tech University, Nanjing 211816, China.

* Correspondence: zyn3648@njtech.edu.cn (Y.Z.); guohu21@njust.edu.cn (H.G.)

1. Adsorption model

1.1. Adsorption kinetics models.

Kinetics models include the pseudo-first-order model, pseudo-second-order model and intraparticle diffusion model. The adsorption equilibrium time and rate-limiting steps of adsorption can be understood by adsorption kinetics to further study the adsorption mechanism. The pseudo-first-order model assumes that the adsorption process is controlled by diffusion [1]:

$$\ln(Q_e - Q_t) = lnQ_e - kt$$

Where Q_t (mg/g) is the adsorption amount of adsorbent at time t (min); Q_e(mg/g) represents the equilibrium adsorption amount; k (min^{-1}) is the pseudo-first-order rate constant.

The pseudo-second-order model assumes that the number of unoccupied vacancies on the adsorbent surface determines the adsorption rate. The adsorption process is mainly chemical adsorption, and the mechanism is mainly related to electron transfer or electron sharing between adsorbent and adsorbent [2]:

$$\frac{t}{Q_t} = \frac{1}{k_2 Q_e^2} + \frac{t}{Q_e}$$

where k_2 (g/mg/min) is the pseudo-second-order rate constant.

The intraparticle diffusion model is based on the following assumptions: (1) The diffusion resistance of the liquid film can be ignored, or the diffusion resistance of the liquid film only exists at the initial stage of adsorption within a short time; (2) The adsorbent diffusion direction is random, and the concentration does not change with the location of the adsorbent; (3) The internal diffusion coefficient is constant and does not change upon changing adsorption time or position. The intraparticle diffusion model is the best kinetic model for describing the diffusion process of the material inside the adsorbent, but it is not suitable for describing the adsorbent on the surface and liquid membrane diffusion process. If Q_t and $t^{1/2}$ data are plotted, a straight line is obtained, which passes through the origin, indicating that the internal diffusion process of the adsorbent is the adsorption--controlling step. If not, the adsorption process is affected by multiple adsorption processes; however, due to the difference in material transfer between the initial stage and the final stage of adsorption, the experimental data generally do not pass through the origin [3].

$$Q_t = k_i t^{\frac{1}{2}} + c$$

where k_i is the intramolecular diffusion constant, and c (mg/g) is a constant.

1.2. Adsorption isotherm.

The adsorption isotherm can be used to describe the relationship between the molecular weight of the solute adsorbed and the concentration of the solute molecule in the solution at the adsorption equilibrium at the same temperature. From the adsorption isotherm, the maximum adsorption capacity of the adsorbent for the pollutant can be calculated, the type of adsorption can be determined, and the strength and ease of adsorption can be understood. The Langmuir and Freundlich models are commonly used for liquid-phase adsorption.

The Langmuir isotherm equation assumes that the surface of the adsorbent is uniform, there is no interaction between the adsorbents, and the adsorbed particles are completely independent. Adsorption proceeds via monolayer adsorption, that is, adsorption occurs only on the outer surface of the adsorbent and involves both chemical and physical adsorption [4].

$$\frac{C_e}{Q_e} = \frac{1}{Q_{\max}K_L} + \frac{C_e}{Q_{\max}}$$

where C_e (mg/L) is the solute concentration at equilibrium; Q_{max} (mg/g) is the maximum adsorption amount; K_L (L/mg) is the Langmuir adsorption constant, which is related to the affinity of adsorbent binding sites. The larger K_L is, the more favorable adsorption is.

The Freundlich isotherm equation can be used for both monolayer and heterogeneous surface adsorption. As a classical formula for heterogeneous surface adsorption, it describes the adsorption mechanism of heterogeneous surfaces, and is also applicable for low-concentration adsorption and can explain experimental results over a wider concentration range; however, the disadvantage of the Freundlich adsorption equation is that the maximum adsorption capacity cannot be fitted and the adsorption action outside the concentration range cannot be estimated. It can be used for both chemical and physical adsorption [5].

$$lnQ_e = lnK_F + ln\frac{C_e}{n}$$

where K_F and n in the formula are Freundlich constants; K_F is related to the adsorption affinity of the adsorbent, and n represents the force between the adsorbent and the adsorbent. When $0 < 1/n < 1$, adsorption occurs easily, and when $1/n > 1$, adsorption is more difficult.

1.3. Adsorption thermodynamics.

Thermodynamics parameters include the Gibbs free energy change ($\Delta G°$), enthalpy change ($\Delta H°$) and entropy ($\Delta S°$). The thermodynamic parameters can be used to judge whether adsorption can proceed spontaneously, the heat absorption/exothermic situation of the adsorption process, and change in the disorder in the adsorption system [6].

$$\ln\left(\frac{Q_e}{C_e}\right) = -\frac{\Delta H^{\circ}}{RT} + \frac{\Delta S^{\circ}}{R}$$

$$\Delta G^{\circ} = \Delta H^{\circ} - T\Delta S^{\circ}$$

where T (K) is the absolute temperature; R (8.314 J/mol K) is the gas constant; $\Delta G°$ (kJ/mol) is the Gibbs free energy change, where $\Delta G° > 0$ indicates a spontaneous reaction; $\Delta H°$ (J/mol) is the enthalpy change, where $\Delta H° > 0$ indicates that the reaction is endothermic; $\Delta S°$ (J/mol K) is the reaction system entropy change, where $\Delta S° > 0$ suggest that the entropy increases.

References

1. Rengaraj S., Kim Y., Joo C. K., Yi J. Removal of copper from aqueous solution by aminated and protonated mesoporous aluminas: kinetics and equilibrium. *Journal of Colloid & Interface Ence* **2004**, *273*, 14-21.
2. Aksu Z., Tezer S. Biosorption of reactive dyes on the green alga Chlorella vulgaris. *Process Biochem.* **2005**, *40*, 1347-1361.

3. Dm H. A., Bulut Y. A kinetics and thermodynamics study of methylene blue adsorption on wheat shells. *Desalination: The International Journal on the Science and Technology of Desalting and Water Purification* **2006**, *194*, 259-267.
4. Langmuir I. The constitution and fundamental properties of solids and liquids. Part II.—Liquids. *J. Am. Chem. Soc.* **1917**, *39*, 1848-1906.
5. Ng C., Losso J. N., Marshall W. E., Rao R. M. Freundlich adsorption isotherms of agricultural by-product-based powdered activated carbons in a geosmin-water system. *Bioresour Technol* **2002**, *85*, 131-135.
6. Nugent P., Belmabkhout Y., Burd S. D., Cairns A. J., Luebke R., Forrest K., Pham T., Ma S., Space B., Wojtas L., Eddaoudi M., Zaworotko M. J. Porous materials with optimal adsorption thermodynamics and kinetics for CO_2 separation. *Nature* **2013**, *495*, 80-84.
